# Examining reactivity to intensive longitudinal ecological momentary assessment: 12-month prospective study

**DOI:** 10.1007/s40519-023-01556-1

**Published:** 2023-02-27

**Authors:** Maan Isabella Cajita, Stephen L. Rathbun, Saul Shiffman, Christopher E. Kline, Christopher C. Imes, Yaguang Zheng, Linda J. Ewing, Lora E. Burke

**Affiliations:** 1 College of Nursing, University of Illinois Chicago, 845 S. Damen Ave., Chicago, IL 60612 USA; 2 Department of Epidemiology & Biostatistics, University of Georgia, Athens, USA; 3 School of Medicine, University of Pittsburgh, Pittsburgh, USA; 4 Department of Health and Human Development, University of Pittsburgh, Pittsburgh, USA; 5 School of Nursing, University of Pittsburgh, Pittsburgh, USA; 6 Rory Meyers College of Nursing, New York University, New York, USA; 7 Department of Epidemiology, University of Pittsburgh, Pittsburgh, USA

**Keywords:** Ecological momentary assessment, Eating behaviors, Self-assessment, Reactivity, Standard behavior treatment

## Abstract

**Purpose:**

To examine the association between intensive, longitudinal ecological momentary assessment (EMA) and self-reported eating behaviors.

**Methods:**

Secondary analysis of the EMPOWER study—a 12-month observational study that examined the microprocesses of relapse following intentional weight loss using smartphone-administered EMA—was conducted. Participants were asked to complete four types of EMA surveys using a mobile app. For this analysis, only the number of completed random EMA surveys was used. Using linear mixed-effects modeling, we analyzed whether the number of completed random EMA surveys was associated with changes in self-reported dietary restraint, dietary disinhibition, and susceptibility to hunger measured using the Three-Factor Eating Questionnaire (TFEQ).

**Results:**

During the 12-month study, 132 participants completed a mean of 1062 random EMA surveys (range: 673–1362). The median time it took for participants to complete random EMA surveys was 20 s and 90% of random EMA surveys were completed within 46 s. The number of completed random EMA surveys was not significantly associated with the TFEQ scores.

**Conclusions:**

Intensive longitudinal EMA did not influence self-reported eating behaviors. The findings suggest that EMA can be used to frequently assess real-world eating behaviors with minimal concern about assessment reactivity. Nonetheless, care must be taken when designing EMA surveys—particularly when using self-reported outcome measures.

**Level of evidence:**

Level III, prospective observational study.

**Supplementary Information:**

The online version contains supplementary material available at 10.1007/s40519-023-01556-1.

The use of ecological momentary assessment (EMA)—the repeated collection of individuals’ experiences, behaviors, and/or moods as they occur in real-world settings—has been on a steady rise over the last decade [1]. The increasing use of EMA as a data collection method can be attributed to the perceived value of ecologically valid, real-time data and the widespread use of smartphones and wearable sensors. However, while technology has enabled EMA to investigate more nuanced associations and processes, the increased frequency of assessments could inadvertently influence the behavior being studied and potentially bias the research findings.

Assessment reactivity refers to the phenomenon in which the action of assessing a behavior independently impacts the expression of that behavior regardless of other interventions used in the study [2]. Assessment reactivity poses a major threat to a study’s internal validity, especially in studies wherein outcomes are assessed solely through self-report [2]. Despite the proliferation of EMA-based studies, the investigation into the impact of frequent assessment on self-reported outcomes is relatively understudied [3–5].

This study aimed to examine the association between intensive, longitudinal EMA—which among other things asked if the participant was eating, if their eating was planned, and if any disinhibiting stimuli were present— and self-reported eating behaviors as measured by the Three-Factor Eating Questionnaire (TFEQ), which assesses disinhibition, restraint, and hunger. If EMA leads to assessment reactivity, then it could be expected that the number of completed EMA surveys will be correlated with the change in TFEQ scores. To test this hypothesis the current study analyzed data from the EMPOWER study, a 12-month observational study that examined the microprocesses of lapse and relapse following intentional weight loss with the use of smartphone-administered EMA.

## Method

### Study design and sample

The current study is a secondary analysis of EMPOWER, a prospective observational study that included 12 months of standard behavioral treatment for weight loss to provide context for observing the triggers of lapses/relapses following intentional weight loss [1]. Standard behavioral treatment consisted of 24 group sessions, daily dietary goals, weekly physical activity goals, and daily self-monitoring (dietary intake, physical activity, and weight). Participants were asked to complete four types of EMA surveys using a mobile app throughout the 12-month study period. First, participants were instructed to initiate an EMA survey if they experienced a lapse or an urge to overeat or eat a food that resulted in their exceeding their calorie or fat goal for the day. Second, participants received an EMA prompt at the beginning of the day, which assessed the previous night’s sleep quality, current mood, daily physical activity and diet goals, and self-efficacy for adhering to the lifestyle plan that day. Third, they received an EMA prompt at the end of the day, which reviewed the day’s activities and general mood. Fourth, participants received either one, two, or four random EMA prompts per day. The random EMA survey included the following questions: (1) where are you?, (2) are you…completely alone (not in view of others)/alone but others are nearby/with others?, (3) are others who are with you eating?, (4) can you see anyone eating?, (5) are you eating?, (6) was this planned?, (7) how are you currently feeling? (response includes checklist of moods including hungry), (8) are you feeling in control of important things? and (9) how confident are you that if you have an urge to go off your healthy lifestyle plan, you can resist the urge? For this paper, only the number of completed random EMA surveys were used in the analysis since their content was more aligned with the questions in the TFEQ and their completion is externally imposed by the study assessment protocol, unlike the self-initiated EMA surveys that are similar to self-monitoring and whose frequency could be related to outcomes. Moreover, unlike beginning of day and end of day assessments which were administered daily, frequencies of random EMAs were randomly manipulated by the study investigators, and so, are expected to show greater variability among participants than the former, and hence greater statistical power for detecting significant evidence of reactivity.

EMPOWER was approved by the University of Pittsburgh Institutional Review Board and written informed consent was obtained from all participants. Individuals were eligible to participate in the EMPOWER study if they met the following inclusion criteria: (1) were ≥ 18 years of age; (2) had a body mass index between 27 and 44 kg/m^2^; and (3) had not participated in another weight loss program in the previous 3 months. Individuals were excluded if they: (1) had any current conditions that may confound study findings (e.g., diabetes, pregnancy, post-bariatric surgery); (2) planned to become pregnant in the next 12 months; (3) planned frequent travel, extended vacations, or relocation in the next 12 months; (4) had a major psychiatric disorder (e.g., schizophrenia); (5) consumed ≥ four alcoholic drinks/day; or (6) were unable or unwilling to use the smartphone.

### Measures

Information on age, sex, race, education level, marital status, and employment status were collected at baseline using a standard questionnaire. The random EMA surveys are available as Supplementary Material. Briefly, the random EMA survey used skip logic and had a minimum of six questions and a maximum of nine. The random EMA survey queried the participants on whether they experienced a lapse or a temptation to eat, the associated triggers, their current mood, and their perceived control over their current situation.

The primary outcomes were the three summary measures of the TFEQ that were administered at baseline, 6 months, and 12 months. The TFEQ is a 51-item questionnaire that measures three distinct eating behaviors: dietary restraint (21 items)—the tendency to deliberately restrict or control food intake; disinhibition (16 items)—the tendency to overeat in the presence of disinhibiting stimuli; and susceptibility to hunger (14 items)—food consumption in response to perceived hunger triggered by internal or external cues [6]. Item responses are scored either 0 or 1 and then summed for each subscale. Higher scores indicate higher levels of dietary restraint, disinhibition, and predisposition to hunger [6]. The TFEQ has demonstrated acceptable to excellent internal consistency (Cronbach’s alpha ranging from 0.79 to 0.92) [6]. For the current study, the Cronbach’s alpha for the restraint subscale was 0.73, 0.62, and 0.66 at baseline, 6, and 12 months, respectively. For the disinhibition subscale, the Cronbach’s alpha was 0.65, 0.75, and 0.79 at baseline, 6, and 12 months, respectively. Finally, the Cronbach’s alpha for the hunger subscale was 0.75, 0.74, and 0.80 at baseline, 6, and 12 months, respectively.

### Statistical analyses

We used linear mixed-effects models to estimate mean TFEQ scores as functions of sampling time (baseline, 6 months, 12 months). Tukey’s studentized range test (HSD) was used to compute confidence intervals for pairwise comparisons among the baseline, 6-month, and 12-month means. The total number of completed EMA surveys was then aggregated across the first 6-month (baseline to 6 months) and second six-month (6 months to 12 months) intervals. Likewise, changes in TFEQ scores (6 months minus baseline, 12 months minus 6 months) were computed for each interval. Two linear mixed effects models with random subject effects were used to test whether the number of completed EMA surveys was associated with changes in each TFEQ score. The first model included the study interval and the number of EMAs completed in that interval, while the second model added an interaction between the study interval and the number of completed EMAs. We chose to model the data over the two time-intervals rather than aggregating the data over the 12-month study period to avoid loss of information that would be available from the former. Having the interaction term allows for the determination if reactivity declined over time as participants habituated to EMAs. All mixed-effects models were fit using SAS 9.4.

## Results

A total of 151 participants enrolled in the EMPOWER study. However, one did not complete any EMAs, seven were lost to follow-up, five withdrew for medical reasons, four withdrew for personal reasons, and three became pregnant. Data from the remaining 132 participants were analyzed. The sample was predominantly female (90%), white (81%), currently married (56%), college-educated (72%), and had a mean age of 50.6 (10.5) years.

During the 12-month study, participants completed a mean of 1062 random EMA surveys (range: 673–1362)—a mean of 526 (SD = 93) in the first 6 months and a mean of 537 (SD = 101) in the second 6 months. The median time it took for participants to complete random EMA surveys was 20 s and 90% of random EMA surveys were completed within 46 s.

The results of the linear mixed effect model (Table 1) showed that there were statistically significant changes in the mean TFEQ scores for dietary restraint (p < 0.0001), disinhibition (p < 0.0001), and susceptibility to hunger (p = 0.01) over the 12-month study. Restraint scores were estimated to be 5.0 (95% confidence interval, 3.9, 6.0) and 4.0 points (95% CI 2.9, 5.0) higher at 6 and 12 months, respectively, than at baseline, but there were no significant differences in restraint between 6 and 12 months. Disinhibition was lower at 6 and 12 months when compared to baseline, but, again, their differences between 6 and 12 months were not significant. Hunger scores decreased from baseline to 6 months, but hunger scores at 12 months did not differ significantly from those at baseline or 6 months.

**Table 1:** Changes in three-factor eating questionnaire (TFEQ) scores over 12 months

Outcome	Means			p-value	Differences between means		
	Baseline	6 months	12 months		B-6mo	B-12mo	6-12mo
Restraint	10.6	15.5	14.5	< 0.0001	− 5.0 (− 6.0, − 3.9)	− 4.0 (− 5.0, − 2.9)	1.0 (− 0.1, 2.0)
Disinhibition	9.7	7.9	8.4	< 0.0001	1.9 (0.9, 2.9)	1.4 (− 0.4, 2.4)	− 0.5 (− 1.5, 0.5)
Hunger	6.3	5.0	5.4	0.0105	1.2 (0.3, 2.2)	0.9 (− 0.1, 1.9)	− 0.4 (− 1.3, 0.6)

95% confidence intervals are provided in parentheses

Table 2 shows the results of fitting linear mixed effects models for the impact of the number of completed EMA surveys on changes in restraint, disinhibition, and hunger scores. No statistically significant interactions were found between interval and completed EMAs for restraint (p = 0.13), disinhibition (p = 0.62), and hunger (p = 0.99). In models excluding the interaction between interval and completed EMAs, the number of completed EMAs had no significant association with restraint (p = 0.96), disinhibition (p = 0.20), and hunger (p = 0.67).

**Table 2:** Results of the linear mixed-effects modeling—random EMAs completed

Source	Restraint		Disinhibition		Hunger	
	Coefficient	p-value	Coefficient	p-value	Coefficient	p-value
Interaction excluded	− 0.01 (− 0.46, 0.43)	0.96	− 0.21 (− 0.55, 0.12)	0.20	− 0.07 (− 0.41, 0.26)	0.67
Interaction included		0.13		0.62		0.99
B-6mo	− 0.38 (− 1.03, 0.27)		− 0.31 (− 0.80, 0.18)		− 0.07 (− 0.57, 0.42)	
6-12mo	0.39 (− 0.30, 0.91)		− 0.14 (− 0.59, 0.32)		− 0.07 (− 0.52, 0.39)	

Rates at which changes in TFEQ scores are estimated to change in each 6-month interval as a function of the number of random EMAs completed (in 100 s) under mixed-effects models excluding and including interactions between number of random EMAs and study interval

Sensitivity analysis was performed using (1) the percentage of random EMA surveys completed (Table S1) and (2) the total number of random EMA prompts (Table S2) instead of the number of completed random EMA surveys in the linear mixed-effects models. The results of the sensitivity analysis—which is available as Supplementary Information—support the original findings; in other words, the percentage of random EMAs completed or the total number of random EMA prompts was not significantly associated with the changes in TFEQ scores.

## Discussion

The interest in real-time behavioral processes and the ubiquity of smartphones have fueled the increased use of EMA in behavioral research. However, despite the proliferation of EMA, only a few studies have examined the unintended effect of intensive longitudinal assessments on behavioral outcomes [3–5, 7, 8]. The results from our secondary analysis of the EMPOWER study showed that despite the significant changes in TFEQ scores over time, these changes were not significantly associated with the number of completed EMA surveys; in other words, assessment reactivity was not observed. To determine the robustness of our findings, we performed sensitivity analysis, which also showed that intensive, longitudinal EMAs did not result in assessment reactivity.

Stein and Corte [3] reported similar findings in their study of women with an eating disorder, wherein EMA frequency was not associated with the frequency of eating disorder behaviors (vomiting, laxative use, diuretic use, exercising, and binge eating). Another study also observed no association between EMA and the number of daily binge eating episodes [4]. Similarly, Heron and Smyth [5] reported that self-reported measures of body image and negative mood were not altered by completing EMAs.

Conversely, studies on smokers seeking to quit reported significant associations between use of EMA and self-reported smoking-related constructs (i.e., craving, anxiety, mood/affect, self-efficacy) but not between EMA and actual smoking cessation [7, 8]. Lastly, Hufford et al. [9] reported no significant association between EMA and changes in alcohol intake.

Overall, whether EMA leads to assessment reactivity appears to depend on the behavior or construct being assessed—with studies on smoking-related constructs finding some evidence of reactivity while studies around eating behaviors finding none. It is worth noting that EMA has not been shown to significantly impact actual behavior such as smoking and drinking [7–10].

This study has several strengths. To the best of our knowledge, this is the first study to examine the impact of intensive EMA over the course of 12 months. Previous studies had much shorter study durations, ranging from 1 week [4, 8] to 4 weeks [3, 7]. Additionally, the study had a high retention rate (87.4%) as well as excellent adherence (88.3%) to completing EMA surveys [1]. Participants also discontinued or abandoned less than 1% of the surveys that they started [1]. Nonetheless, findings from this secondary analysis should be interpreted within the context of its limitations, including the study sample being predominantly female, White, and well-educated, whether our findings can be generalized to males, other races, and those of lower educational attainment is uncertain. We did not assess reactivity over a shorter period. Lastly, all the participants received standard behavior treatment for weight loss; hence, our findings may not extend to non-intervention studies.

## Conclusions

Findings from this study have important implications for behavioral and clinical research. This secondary analysis found no significant associations between longitudinal intensive EMA and self-reported measures. Our findings suggest that EMA can be used to frequently assess real-world eating behaviors with minimal concern that it will lead to assessment reactivity. Nonetheless, care must be taken when designing EMA surveys—particularly when using self-reported outcome measures.

## Strengths and limits

- First to study the impact of intensive EMA over a prolonged time period (12 months)
- The EMPOWER study had a high retention rate (87.4%), and
- High completion rate for random EMAs (88.3%)
- Predominantly White (81%) and female (90%) study sample
- Assessment reactivity over a shorter period was not evaluated

## What is already known on this subject?

Previous studies on eating disorder behaviors (vomiting, laxative use, diuretic use, exercising, and binge eating) and body image showed that EMAs did not lead to assessment reactivity.

## What this study adds?

The EMPOWER study is the first to examine the impact of intensive EMA (up to 4 random EMAs daily) on self-reported dietary restraint, disinhibition, and perceived hunger over a prolonged period.

## Supplementary Information

Below is the link to the electronic supplementary material.

Supplementary file 1 (DOCX 26 KB)

## Data Availability

The dataset analyzed during the current study is available from the corresponding author on reasonable request.
