# Supplementary material for: Examining reactivity to intensive longitudinal ecological momentary assessment: 12-month prospective study
Source: Eat Weight Disord. 2023 Feb 27;28(1):26. doi: 10.1007/s40519-023-01556-1 (PMC9971140; doi:10.1007/s40519-023-01556-1)
Supplement: Supplementary file 1 — (DOCX 26 KB) [file 40519_2023_1556_MOESM1_ESM.docx]

**Supplementary Information: Results of the Sensitivity Analysis**

| **Table S1. Results of the Linear Mixed-Effects Modeling – Percent of Random EMAs Completed** | | | | | | |
| --- | --- | --- | --- | --- | --- | --- |
|  | **RESTRAINT** | | **DISINHIBITION** | | **HUNGER** | |
| Source | Coefficient | p-value | Coefficient | p-value | Coefficient | p-value |
| Interaction Excluded | 0.004  (-0.047, 0.054) | 0.87 | 0.011  (-0.027, 0.049) | 0.57 | -0.005  (-0.044, 0.033) | 0.78 |
| Interaction Included |  | 0.77 |  | 0.90 |  | 0.52 |
| B-6mo | 0.014  (-0.069, 0.096) |  | 0.008  (-0.054, 0.070) |  | 0.011  (-0.051, 0.073) |  |
| 6-12mo | -0.002  (-0.066, 0.062) |  | 0.0129  (-0.035, 0.061) |  | -0.015 (-0.063, 0.033) |  |
| *Rates at which changes in TFEQ scores are estimated to change in each 6-month interval as a function the percent of random EMAs completed under mixed-effects models excluding and including interactions between percent of random EMAs and study interval.* | | | | | | |

| **Table S2. Results of the Linear Mixed-Effects Modeling – Total Number of Random EMA Prompts** | | | | | | |
| --- | --- | --- | --- | --- | --- | --- |
|  | **RESTRAINT** | | **DISINHIBITION** | | **HUNGER** | |
| Source | Coefficient | p-value | Coefficient | p-value | Coefficient | p-value |
| Interaction Excluded | -0.01 (-0.48, 0.47) | 0.87 | -0.31 (-0.67, 0.04) | 0.08 | -0.06 (-0.41, 0.30) | 0.73 |
| Interaction Included |  | 0.08 |  | 0.67 |  | 0.68 |
| B-6mo | -0.46 (-1.16, 0.23) |  | -0.40 (-0.92, 0.13) |  | -0.14 (-0.66, 0.39) |  |
| 6-12mo | 0.39 (-0.25, 1.02) |  | -0.24 (-0.72, 0.23) |  | -0.01(-0.47, 0.49) |  |
| *Rates at which changes in TFEQ scores are estimated to change in each 6-month interval as a function of the number of random EMA prompts (in 100s) under mixed-effects models excluding and including interactions between number of random EMAs and study interval.* | | | | | | |
